# Cellular sources of TSPO expression in healthy and diseased brain

**DOI:** 10.1007/s00259-020-05166-2

**Published:** 2021-01-12

**Authors:** Erik Nutma, Kelly Ceyzériat, Sandra Amor, Stergios Tsartsalis, Philippe Millet, David R. Owen, Vassilios Papadopoulos, Benjamin B. Tournier

**Affiliations:** 1 Department of Pathology, Amsterdam UMC, VUmc, Amsterdam, The Netherlands; 2 Division of Adult Psychiatry, Department of Psychiatry, University Hospitals of Geneva, Avenue de la Roseraie, 64, 1206 Geneva, Switzerland; 3 Division of Nuclear medicine and Molecular Imaging, University Hospitals of Geneva, Geneva, Switzerland; 4 Division of Radiation Oncology, Department of Oncology, University Hospitals of Geneva, Geneva, Switzerland; 5 Centre for Neuroscience and Trauma, Blizard Institute, Barts and the London School of Medicine & Dentistry, Queen Mary University of London, London, UK; 6 Department of Brain Sciences, Faculty of Medicine, Imperial College London, London, UK; 7 Department of Psychiatry, University of Geneva, Geneva, Switzerland; 8 Department of Pharmacology and Pharmaceutical Sciences, School of Pharmacy, University of Southern California, Los Angeles, CA USA

**Keywords:** TSPO, Astrocytes, Microglia, Positron emission tomography

## Abstract

The 18 kDa translocator protein (TSPO) is a highly conserved protein located in the outer mitochondrial membrane. TSPO binding, as measured with positron emission tomography (PET), is considered an in vivo marker of neuroinflammation. Indeed, TSPO expression is altered in neurodegenerative, neuroinflammatory, and neuropsychiatric diseases. In PET studies, the TSPO signal is often viewed as a marker of microglial cell activity. However, there is little evidence in support of a microglia-specific TSPO expression. This review describes the cellular sources and functions of TSPO in animal models of disease and human studies, in health, and in central nervous system diseases. A discussion of methods of analysis and of quantification of TSPO is also presented. Overall, it appears that the alterations of TSPO binding, their cellular underpinnings, and the functional significance of such alterations depend on many factors, notably the pathology or the animal model under study, the disease stage, and the involved brain regions. Thus, further studies are needed to fully determine how changes in TSPO binding occur at the cellular level with the ultimate goal of revealing potential therapeutic pathways.

## Introduction

The 18 kDa translocator protein (TSPO) is increasingly used as a marker for in vivo neuroinflammation with positron emission tomography (PET) in a wide variety of CNS diseases. Many of these studies reveal that TSPO PET signal is altered in neurodegenerative, neuroinflammatory, and neuropsychiatric diseases when compared to healthy individuals [1]. Historically, TSPO has been viewed as a marker of microglial cell activity. Indeed, the first studies showing a correlation between the binding of one of the TSPO tracers and microglia speculated that TSPO is a marker for pathogenic microglial cells [2, 3]. This idea is no longer maintained. Indeed, the presence of TSPO in other cell types, notably astrocytes and endothelial cells, has been demonstrated [4–7].

TSPO is not the only molecular target for PET imaging of neuroinflammation. Several other receptors that are expressed in brain cells and are potentially upregulated in neuroinflammation have been identified and specific radiotracers are developed to label them in vivo. For instance, radiotracers binding the cannabinoid receptor 2 (CB_2_) receptor [8, 9] and cyclooxygenase-2 (COX-2) [10, 11] have been validated in preclinical models of brain disease and generally showed a robust increase in binding associated with neuroinflammation, while the first results from human PET studies are encouraging [12, 13]. In addition, radiotracers binding the purinergic receptor subtype 7 (P2X7) [14–16], the sphingosine-1-phosphate receptor 1 (S1PR1) [17], reactive oxygen species [18], and the colony stimulating factor 1 receptor (CSF1R) [19] are at initial stages of preclinical validation. In light of these results, it is clear that TSPO is the most extensively studied molecular target for in vivo PET imaging of neuroinflammation to date.

Here we review the cellular sources and functions of TSPO in animal models and humans in health and in CNS diseases. This will contribute to a better understanding of the function of TSPO, the physiology of TSPO expression, and its functional consequences in the human body and the CNS. Furthermore, knowledge about the expression of TSPO in CNS diseases provides insight into expression patterns and its predictive potential in diagnosing CNS diseases and disease progression with TSPO PET.

## TSPO functions in health

TSPO participates in many essential mitochondria-based physiological processes, including metabolism and cellular bioenergetics, mitochondrial respiration, cholesterol transport and steroidogenesis, immunomodulation, porphyrin transport, and heme biosynthesis [20–24]. It has also been suggested that TSPO may play critical roles in cell proliferation, tumorigenesis, and apoptosis [24–26]. In order to discern and evaluate the function of any protein, particularly one like TSPO that seems to be multifunctional, one must consider specific characteristics that could provide clues to the possible roles it may play. The characteristics that need to be investigated should include its (i) tissue, cellular, and subcellular localization, (ii) characteristics and effects of endogenous and exogenous ligands, (iii) molecular structure and cellular functions, (iv) genetics and genetic models, and (v) evolution.

### Tissue, cellular, and subcellular localization

TSPO was first characterized for its ability to bind with specificity and high-affinity various classes of chemicals such as benzodiazepines, isoquinoline carboxamides, indole acetamides, pyrazolopyrimidines, and aryloxyanilides, as well as endogenous ligands including porphyrins, the endozepine diazepam binding inhibitor [20–24, 27–33]. Radioligand binding and later on immunodetection studies revealed that the distribution pattern of TSPO between rodents and humans is similar; secretory and glandular tissues were particularly rich in TSPO. These studies also indicated that although TSPO is present in most tissues in most species at various levels of expression, it is most abundant in steroid-synthesizing adrenal and gonadal tissues. The heart and kidney express intermediate levels of TSPO, while lower levels are found in the liver and brain. Interestingly, when considering the mitochondrial content of each tissue, there is not always a clear correlation between TSPO levels and mitochondrial content. This finding suggests that tissue- and cell-specific factors regulating *TSPO* gene expression are driving TSPO content rather than factors driving mitochondria formation.

Steady-state mRNA profiling shows that *TSPO* mRNA is present in all tissues and correlates well with reported protein expression levels [25]. Moreover, the expression patterns of mouse TSPO were found to be well-correlated and similar to that reported for human *TSPO* [34, 35].

TSPO levels were found to be elevated in cancer cell lines and numerous cancers suggesting a role for TSPO in cell proliferation and carcinogenesis [24, 25, 29, 36–38]. Increased TSPO levels in cancer are due to gene amplification; Sp1, Sp3, and Sp4 transcription factor regulation of constitutive TSPO expression; and epigenetic modifications of the proximal promoter and first intron [25, 39, 40].

TSPO is an integral outer mitochondrial membrane protein spanning the membrane through its 5 α-helical domains [41–54]. While TSPO is a nuclear encoded protein, unlike most mitochondrial proteins, TSPO does not possess a mitochondrial targeting sequence, although it contains information on the C-terminus that is essential for its mitochondrial import [55]. After integration into the OMM, TSPO forms dimers and sometimes polymers [20, 56, 57] at the outer and inner mitochondrial membrane contact sites where it becomes part of a larger protein complex [58]. This complex includes the OMM voltage-dependent anion channel 1 (VDAC1); ATPase family AAA domain-containing protein 3 (ATAD3), a protein that spans across the mitochondrial membranes, and in steroidogenic cells; and the inner mitochondrial membrane cytochrome P450 side-chain cleavage enzyme (CYP11A1), among others [58, 59]. In addition, cytosolic, endoplasmic reticulum and Golgi proteins have been shown to associate with TSPO to form functional complexes [59–61]. When assembled together, these proteins function as a signal transduction complex, or “transduceosome” mediating the transmission of information to mitochondrial TSPO.

Although 95% of TSPO is found in the mitochondria, the protein can be found in intracellular locations other than mitochondria, such as the (peri)nuclear region and plasma membrane, likely playing different functions. Nevertheless, non-mitochondrial TSPO [43, 62] has received little attention so far.

### Characteristics and effects of endogenous and exogenous ligands

TSPO is involved primarily in the mitochondria of steroid synthesizing cells. Steroidogenesis in the mitochondria begins with the transport of substrate cholesterol from intracellular stores into the mitochondria. Therefore, the role or roles of TSPO in mitochondrial steroidogenesis and cholesterol transport, in particular, were investigated.

With the availability of high-affinity TSPO ligands, the function of TSPO in various tissues was explored, aiming to assess whether these ligands could affect mitochondrial function, including steroid production. Several TSPO ligands were found to affect mitochondrial respiration [63] and increase oxygen consumption [64] and ATP synthesis [65]. At the same time, detailed studies demonstrated the ability of these ligands to induce cholesterol transport into mitochondria and steroid formation in all steroidogenic cells in vitro and in vivo [56, 66, 67]. These studies were later extended to neurosteroid synthesizing glia cells in the brain [68–71]. TSPO ligands were also shown to affect intracellular cholesterol trafficking and lipid droplet accumulation, a function that may not be related to steroidogenesis [72, 73].

However, there are ligand-specific differences as well as off-target effects. These differences may be explained by the tissue and cell-specific microenvironment and the presence of endogenous ligands, e.g., porphyrins and endozepines, in specific tissues/cells that may compete with the exogenous ligand. Moreover, the fact that TSPO exists within large protein complexes suggests that TSPO ligand selectivity may be governed by the protein-complex composition and not only by the interaction with TSPO alone [74]. In addition, it was recently shown that TSPO ligands have different occupancy times for TSPO and this affects their ability to induce steroid formation [75, 76]. Concerning the off-target effects, most of the time these are linked to the use of high concentrations of TSPO ligands, thousands of time higher than the affinity of these compounds for TSPO. Indeed, lipophilic TSPO ligands used at high concentrations are likely to interact with membranes or other not yet identified targets resulting in off-target effects [66, 77]. In addition, TSPO ligands were found to exert cell-type specific effects raising again the question of the role of the microenvironment, ligand residence time, and the presence of endogenous ligands [78, 79].

Over the years, the effects of TSPO drug ligands with various mitochondrial activities/functions have also been shown, including changes in VDAC1, F-ATP synthase and ANT activities, modulation of reactive oxygen species (ROS) production, and calcium levels and effects on mitochondrial membrane potential and permeability transition pore (MPTP) [51, 52, 59, 80–85]. These effects were found to be tissue- and cell-specific and sometimes ligand-specific or observed only in cell lines. However, in some cases, the effects were observed in the presence of micromolar concentrations of TSPO ligands, far beyond the affinity of the protein for the compounds. The complex formed by the mitochondrial TSPO in association with VDAC1 has been suggested to have a role in apoptosis, possibly through MPTP opening, and cholesterol transport. TSPO drug ligands have been found to exert both proliferative and antiapoptotic effects, as well as antiproliferative properties, acting in a biphasic manner [24, 25, 29, 36–38, 86].

### Molecular structure and cellular functions

The drug ligand binding domains of TSPO have been mapped [87] and it was subsequently shown that TSPO is a high-affinity cholesterol binding protein containing a conserved cholesterol recognition amino acid consensus domain in the C-terminus [88, 89]. The drug and cholesterol binding domains were found to be in distinct domains of the protein results confirmed by NMR [87, 88, 90]. Moreover, these findings were further confirmed in structural studies by NMR and crystallography studies that reported the atomic structure of TSPO [91–96]. These studies also proposed that the functional TSPO is a dimer, that ligand binding to TSPO can promote cholesterol movement, and that cholesterol is an allosteric regulator of TSPO [91, 93, 94, 97].

The ability of TSPO to bind drug ligands and cholesterol is its two major intrinsic properties and mostly likely the ones determining its function. We summarized above the reported effects of TSPO ligands on mitochondrial function. Although in steroidogenic and liver cells the role of a cholesterol binding protein segregating the steroidogenic pool of cholesterol from structural cholesterol and facilitating its import into the mitochondria for steroid and formation is obvious, for other cells it is not so clear. However, cholesterol transfer in the inner mitochondrial membrane is needed for biogenesis of mitochondrial membranes during cell proliferation and/or repair. TSPO may also function as a sink for cholesterol which when free could be toxic for the cells. It is also possible that TSPO may be facilitating the movement of free cholesterol from the mitochondria to other organelles, as shown in astrocytes [72], fibroblasts [72], macrophages [98], retinal cells [99], and the steroidogenic Leydig cells [73]. Moreover, TSPO-mediated accumulation of free cholesterol in the mitochondria may affect mitochondrial membrane fluidity/permeability, fission/fusion processes, membrane protein/transporter function(s), and/or membrane potential [72, 83, 100–102].

TSPO was also shown to regulate mitophagy [59, 103]. TSPO, by binding to VDAC1, reduces mitochondrial coupling and promotes an overproduction of ROS that counteracts Parkin-mediated ubiquitination of proteins. These data suggested TSPO as an element in the regulation of mitochondrial quality control by autophagy. Further studies showed that TSPO deregulates mitochondrial Ca^2+^ signaling, leading to a parallel increase in the cytosolic Ca^2+^ pools that activate the Ca^2+^-dependent NADPH oxidase, thereby increasing ROS [104]. The inhibition of mitochondrial Ca^2+^ uptake by TSPO is a consequence of the phosphorylation of VDAC1 by PKA, which is recruited to the mitochondria by ACBD3, VDAC1, ACBD3, PKA, and all transduceosome components recruited at TSPO. This is proposed as a novel OMM-based pathway to control intracellular Ca^2+^ dynamics and redox transients in cytotoxicity [104].

### Genetics and genetic models

A series of articles came out in the last 15 years assessing the direct role of TSPO in various cellular pathways. First, the role of TSPO in opening the MPTP in liver mitochondrial function was investigated in an animal model depleted of liver TSPO [105]. The data obtained showed that the absence of TSPO does not affect liver MPTP function. Then, studies in rodents with genetic depletion of *TSPO* led to conflicting results including no effect on steroid synthesis [106–108], reduced steroid output, inhibition of corticosteroid response to adrenocorticotropic hormone, changes in lipid homeostasis in Leydig cells and reduction of circulating testosterone levels, and suppression of neurosteroid formation [109–112].

In addition, discordant data was reported on MA-10 mouse Leydig cells. Knockdown of *TSPO* expression using antisense oligonucleotides or antisense RNA reduces the ability of the cells to form steroids, while CRISPR/Cas9-guided *TSPO* deletion has either no effect or abolishes steroid synthesis [113–116]. These differences have been discussed in detail in other reviews [66, 67].

Among all these studies, it seems that there is consistency between laboratories on the role of TSPO in neurosteroid formation where genetic deletion of TSPO led to reduced neurosteroid synthesis [110, 112]. These results suggest that the role of TSPO in steroid formation may be primary and rate-determining in cells where steroid formation is independent on hormonal control, e.g., brain, compared to the classical peripheral steroid forming gonads and adrenal where pituitary hormones control the massive steroid production. In peripheral steroidogenic organs, TSPO may play a secondary role or play a role in cases where the cells do not respond to pituitary hormones, as in male hypogonadism where TSPO ligands can recover the drug- or age-induced reduction in androgen formation [66].

Numerous biochemical, pharmacological, and clinical data in the field of photodynamic therapy in oncology have demonstrated the role of ability of TSPO to bind porphyrins and its role in porphyrin and heme transport and synthesis [117–119]. Using the same mice as before [107], the same group failed to show a role for TSPO in porphyrin and heme biosynthesis or transport [120].

TSPO deficiency decreased the oxygen consumption rate and mitochondrial membrane potential in mouse fibroblasts [120], MA-10 mouse Leydig cells [116], and C20 human microglia cells where it also reduced respiratory function [121]. Mitochondrial membrane potential depends on the flux of respiratory substrates adenosine triphosphate, adenosine diphosphate, and Pi through VDAC. Adenine nucleotide translocator also plays a role in maintenance of the membrane potential [116]. Therefore, TSPO likely controls cellular and mitochondrial metabolism via regulation of the mitochondrial membrane potential and affects OMM permeability and/or outer and inner membrane contacts/fusion.

Interestingly, lack of TSPO was shown to affect mitochondrial respiration and increase oxygen consumption in some cell and animal *Tspo* KO models, but not in others [63, 65, 79, 107, 108, 120, 121]. More recent studies also failed to show a direct link of TSPO to F-ATP synthase, which was shown to form the MPTP [122].

The differences underlying the disparate results from these genetic animal and cell models are not well understood. However, they clearly indicate differences between the pharmacology of TSPO and its intrinsic cellular functions. It is also likely that species differences, the presence of external or intrinsic stimuli, as well as differences in age, sex, and metabolic status of the species used may control the expression of TSPO. Considering that TSPO is one of the evolutionarily oldest proteins (see below), we proposed that it serves as the basis for fundamental functions and, thus, in case of its absence, compensatory mechanisms may have evolved. Moreover, even if its absence may not always affect animal phenotype, its presence, concentrated at the OMM, plays a regulatory role in mitochondrial function and associated tissue-specific phenotypes. Moreover, its presence provides us with a molecular target able to modulate mitochondrial and cell functions.

TSPO genetics in humans provide some of the most important information on the function of this protein. No humans have been identified lacking TSPO. In humans, the presence of a number of polymorphisms have been identified in the *TSPO* gene, including rs6971 [123]. This polymorphism causes a non-conservative amino acid substitution, Ala147Thr, resulting in altered binding affinity of TSPO for specific ligands [123]. The presence of this *TSPO* polymorphism has been linked to the function of the hypothalamic-pituitary-adrenal axis, predisposing carriers to psychiatric disorders [124–127], and potentially impairing the response of patients to anxiolytic TSPO drug ligands [128, 129]. The presence of this *TSPO* polymorphism was linked to reduced pregnenolone [130] and adrenocorticotropic hormone (ACTH)-induced corticosteroid levels [110] and shown to be associated with dysregulated cortisol rhythms and consequent clinical exacerbations in bipolar disorders [131]. This finding provides clear evidence of the link between TSPO, cholesterol binding, and steroid formation under normal and stress conditions.

### Evolution

TSPO is an evolutionary conserved 3.5-billion-year-old protein [132]. TspO, named for its high tryptophan content and apparent role in the regulation of the transition between photosynthesis and respiration, is the mammalian TSPO ortholog in the photosynthetic bacterium *Rhodobacter* [133], a close living relative of mitochondria [57]. Detailed evolutionary studies indicated that the *Tspo* gene family has been expanded by gene duplications from a bacterial environmental sensor or signal transducer to a functional bioregulator adapted to organism-, tissue-, cell-, and organelle-specific needs. Interestingly, the mammalian protein is able to rescue the phenotypes of bacterial *TspO* KO suggesting a conserved function [134] and that one compensates for the loss of oxygen sensing function that occurs when the other is depleted.

An additional Tspo family member, Tspo2, has been characterized [135]. Comparative analysis of Tspo1, the first family member to be identified, and Tspo2 structure and function indicates that TSPO2 was characterized by the loss of diagnostic drug ligand binding, but retention of cholesterol binding properties, and is involved in cholesterol redistribution during erythropoiesis [135]. Whether there are additional family members in mammals or humans remains to be determined. However, the highly conserved sequence would seem to indicate that such expansion in members was not needed to support the rich expansion of cellular functions.

Pharmacological and structural evidence supports TSPO functioning in tetrapyrrole biosynthesis, porphyrin transport, heme metabolism, cholesterol transport/trafficking, steroid formation, control of ROS levels, and the protection of mitochondria from free radical damage. All evolutionarily conserved functions are linked to mitochondria and affected by changes in mitochondrial membrane potential, a function dependent on the presence of TSPO. Few years ago, we proposed that the central role of TSPO throughout evolution is in oxygen-mediated metabolism. This central function has diversified roles in tissue- and cell-specific signaling, metabolism, cholesterol trafficking, immunological responses, apoptosis, steroid synthesis, and host-defense response to disease and injury, all oxygen-mediated pathways [22, 132].

### Summary

TSPO is a multifunctional protein involved in a wide array of cellular functions that are essential for human health. Its central location in the mitochondria, a multifunctional organelle itself, underscores its importance at the crossroads of critical homeostatic pathways. Its evolutionarily conserved sequence also supports its cellular significance. As we continue to elucidate the intricacies of the role of TSPO in health and disease, we will have the opportunity to identify new therapeutic and diagnostic targets that will have significant impact in the near and long term.

## TSPO cell origin in wild-type and preclinical models of neurological disease

A summary of the cell origin of TSPO according to the pathology is given in Table 1.

**Table 1:** TSPO cell origin in preclinical models of neurological diseases

Human use	Preclinical model	Microglia	Astrocytes	Endothelial cells	References
Acute inflammation	LV-CNTF	x	x		[138]
	AAV-TNF	x	x	x	[139]
Ischemia	MCAO	x	x		[141–143]
Multiple sclerosis	EAE	x			[146, 150]
	EAE in TSPOko		x		[149]
	CPZ	x	x		[147, 148]
Alzheimer’s disease	APP23		x		[153]
	PS19	x			[153]
	APP_swe_/PSEN1_ΔE9_	x	x		[151, 154]
	APP^NL-G-F^	x			[157]
	3xTg-AD	x	x		[159]
	5xFAD	x			[154, 155]
Schizophrenia	MIA	x	x	x	[191]

*LV-CNTF* lentivirus encoding ciliary neurotrophic factor, *AAV-TNF* adeno-associated virus encoding tumor necrosis factor, *MCAO* middle cerebral artery occlusion, *EAE* experimental autoimmune encephalomyelitis, *TSPOko* 18 kDa translocator protein knockout, *CPZ* cuprizone, *APP* amyloid precursor protein, *PSEN* presenilin, *3xTg* triple transgenic model, *5xFAD* five AD-linked mutation model, *MIA* maternal immune activation model

### Wild-type

The density and cell origin of TSPO were assessed in the mouse brain. The specificity of TSPO staining was confirmed using a TSPO-deficient strain [136]. Two major findings were reported: TSPO is not homogeneously expressed in the various brain regions and the cell origin also varies across brain regions. The cerebellum shows high levels of TSPO staining as does the choroid plexus and the ependyma of the ventricular system. In addition, TSPO expression in the white matter is generally higher than in the gray matter. Regarding the cell origin of TSPO, in the cortex, astrocytes and microglia lack the constitutive TSPO expression observed in white matter. In contrast, in the hippocampus, TSPO is predominantly present in the subgranular layer and partially colocalizes with astrocytes but not with microglia. In the cerebellum, Purkinje cells are responsible for the expression of TSPO. Endothelial cells and pericytes of blood vessels also express TSPO. Furthermore, TSPO-positive NG2 cells were found in the spinal cord of mice (Daugherty et al., 2013). Finally, TSPO seems to be absent from neurons and oligodendrocytes in all brain regions. However, a recent study shows a strong colocalization between TSPO and tyrosine hydroxylase (the limiting enzyme of dopamine synthesis) in the substantia nigra [137]. The authors concluded of that study that TSPO is present in the neurons of the dopaminergic system.

### CNTF and TNF

Numerous studies have sought to highlight the alterations in its expression in response to either inflammatory stimuli. A first study evaluated the response to the overexpression of the ciliary neurotrophic factor (CNTF) [138]. The authors administered a lentivirus coding for the CNTF (containing an export sequence to be released outside the cell) via an intracerebral injection to focally induce an artificial expression of the CNTF in the brain. Two to six months after the injection, a significant increase in TSPO binding was observed on the ipsilateral side in PET imaging, which was confirmed ex vivo by western blotting and mRNA quantification. In order to characterize the cells that accounted for this upregulation, double immunofluorescence was performed for TSPO, IBA1 (a marker of microglia), and GFAP (a marker of astrocytes). In the contralateral (vehicle-treated) side, TSPO was found in microglial cells but not in astrocytes. Conversely, on the ipsilateral side, TSPO was localized in microglia as well as astrocytes indicating that CNTF induced TSPO in astrocytes. In contrast, in microglia, it is difficult to conclude whether a modification of the TSPO has taken place or not, in the absence of quantification. Indeed, although the TSPO is present on both sides of the brain (treated and control), it is possible that its level is increased in response to the chronic CNTF exposure. In a second study using a similar approach, an adenovirus encoding the sequence of the tumor necrosis factor (TNF) gene was injected into the mouse brain and analyses were performed at 3 or 5 days post injection [139]. Colocalization studies demonstrated the presence of TSPO in astrocytes and microglia. In addition, compared to the non-injected side, there was an increase in the number of double-positive cells for TSPO with GFAP, CD11b (a microglial marker), as well as CD31 (an endothelial marker), as revealed by flow cytometry.

### Ischemia

One of the most popular animal models of ischemia is achieved by a unilateral occlusion of the middle cerebral artery (MCAO) [140]. This intervention induces a progressive inflammatory reaction that is associated with an increase in TSPO at the mRNA and protein levels [141, 142]. To determine the cellular origin of TSPO in this experimental model, 1 week after a 60-min intraluminal occlusion of MCA, TSPO^+^ labeling was reported in microglial cells (as shown by lectin immunoreactivity) at the site of the core of the ischemia [142]. At the periphery of the ischemic core, some of the GFAP^+^ cells were also TSPO^+^, compared with the contralateral side. Using a similar protocol but with an occlusion time of 90 min, there was a strong TSPO^+^CD11b^+^ colocalization indicative of a microglial origin of TSPO [141]. However, these data are qualitative and an assessment of the colocalization between TSPO and other cell-type markers was not performed. A third study showed an increase in the number of TSPO^+^ cells in response to the occlusion of MCA as demonstrated by cytometry [143]. More precisely, the TSPO^+^ cells expressed microglial markers (Cd11b^+^CD45^int^ or IBA1^+^). Interestingly, pretreatment with the TSPO agonist etifoxine helped to contain the size of ischemia, decreased neurological symptoms, and reduced cytokine release in response to the MCAO. These effects were abolished in a model of ischemia combined with a chemical inactivation of microglia [143]. Thus, it is probable that TSPO from microglia plays a role in inflammation in MCAO models. However, the search for TSPO in other cell types is not constant and will therefore require further investigations to better define, for example, the role of TSPO of astrocytic origin.

### Multiple sclerosis

Experimental autoimmune encephalomyelitis (EAE) and cuprizone (CPZ) intoxication models induce demyelination and proliferation of microglial and astrocytic cells and are thus useful animal models of multiple sclerosis (MS) [144, 145]. An increase in TSPO density has been reported in such animal models, using TSPO radioligands ([^3^H](R)-PK11195, [^18^F]DPA-714, [^18^F]GE180) [146–148]. By assessing the colocalization between TSPO and IBA1 or GFAP after exposure to CPZ (administered *per os* in the animals’ food) for 1, 3 or 5 weeks, virtually all IBA1^+^ cells expressed TSPO in both control and CPZ-treated animal. In contrast, only a small portion of GFAP^+^ were TSPO^+^ positive in control animals, whereas about 35% of GFAP^+^ cells become TSPO^+^ after 5 weeks of treatment. These changes were mainly localized in the *corpus callosum* and, to a lesser extent, in the gray matter cortex [147]. The CPZ models make it possible to investigate the expression of TSPO during the demyelinating phase (during CPZ treatment), as well as during the remyelination phase (after CPZ treatment). Thus, Zinnhardt et al. (2019) used a 4- and a 6-week CPZ treatment to explore both phases of the CPZ treatment [148]. They observed increases in TSPO binding in both states compared with the controls. TSPO levels in the demyelination phase are higher than during the remyelination phase. TSPO expression was mainly microglial during the demyelination phase and both microglial and astrocytic during the remyelination phase. These results were based on the colocalization of TSPO^+^IBA1^+^ and TSPO^+^GFAP^+^, a qualitative finding, without precise quantitative information regarding the relative contribution of these two glial cell types in the alterations in TSPO binding. In a mouse EAE model, photoemulsion of the in vitro binding of [^3^H](R)-PK11195 on brain sections [146] showed that the TSPO radioligand binding was colocalized with OX-42 (a marker of microglia). The authors reported no co-staining between [^3^H](R)-PK11195 and GFAP. However, in a mouse model of specific TSPO deficiency in astrocytes (hGFAP-driven conditional TSPO knockout mice), the astrocytic proliferation and the behavioral signs, both associated with EAE, were milder, compared to wild-type animals [149]. Importantly, treatment with the TSPO ligand etifoxine decreases the severity and increases the symptomatic recovery in a EAE mouse model of MS [150]. These findings suggest that the microglial and astrocytic TSPO differentially contribute to animal models of MS.

### Alzheimer’s disease

Alzheimer’s disease (AD) is characterized by the accumulation of amyloid deposits mainly formed by the beta amyloid peptide (Aβ) and by the presence of neurofibrillary tangles formed from abnormal forms of the Tau protein. Animal genetic models of the disease are produced by the induction of Aβ overexpression (by adding transgenes encoding human forms of APP or PS1) or overexpression of abnormal forms of Tau (by adding transgenes coding for human Tau forms) [151]. In all AD models, an overexpression of TSPO is observed [3, 6, 152–159]. However, there is no consensus on the cellular origin of TSPO [160]. Indeed, in the APP23 transgenic (Tg) mice model, astrocytes represent the cellular source of TSPO expression in the vicinity of extracellular amyloid deposits [153]. In contrast to this model, the PS19 Tg mice show microglia TSPO expression [153]. The presence of a significant spatial correlation between [^3^H](R)-PK11195 binding and IBA1 staining that is not present in the case of GFAP staining suggests that the origin of TSPO is predominantly microglial in the APP_SWE_/PSEN1_ΔE9_ mice model [3]. However, double immunofluorescence revealed that the cellular origin of TSPO may mainly be microglial although TSPO^+^GFAP^+^ cells were also present [154]. The predominance of a microglial origin to the TSPO binding is also observed in the 3xTgAD model (APP_SWE_/PS1_M146V_/Tau_P301L_) with the use of IBA1 and GFAP co-staining with TSPO [159]. In a model combining three APP mutations and two PS1 mutations (5XFAD), colocalization of TSPO with GFAP or S100β for astrocytes is absent while TSPO^+^IBA1^+^ cells are observed. Interestingly, Liu et al. (2005) also reported that subtypes of microglial cells are differentially contributing to the expression of TSPO. Indeed, TSPO strongly colocalizes with the CD68 microglial pro-inflammatory marker. In addition, TSPO is also present in microglia positive for the CD206 anti-inflammatory marker when these cells are in the vicinity of the amyloid deposits [154]. Thus, the complexity of glial cell types and the differential expression of TSPO by the various subclasses of glial cells add another level of complexity that needs to be further studied.

### Schizophrenia

In contrast to the aforementioned pathologies, the density of TSPO is decreased in schizophrenia (see details in the next chapter). Using the maternal immune activation (MIA) animal model of schizophrenia, a decrease in the TSPO levels was reported [161]. The authors observed a decrease in colocalization of TSPO with IBA1, GFAP, and Glut1 (a marker of the brain vasculature) [161], suggesting the involvement of multiple different cell types.

### General considerations

Overall, it is important to discuss several critical issues. First, the assessment of TSPO in other cell types than microglia has not been examined systematically, e.g., in astrocytes and even less in endothelial cells. Secondly, many studies use IBA1 as a marker of microglia, but it represents a ubiquitous labeling of this cell type and not of any specific activated forms or phenotypes. Without a more in-depth analysis of the TSPO^+^IBA1^+^ cells, it cannot be completely affirmed that it is indeed active microglia and even if it is, the pathophysiological significance of a particular phenotype of activated microglia or indeed other cells of the monocyte family that also express IBA1. In this context, several reports using mouse primary glial cell cultures demonstrate that TSPO is more likely to be modified in activated (by pro-inflammatory stimuli) forms of microglia and astrocytes [139, 162, 163]. Finally, studies of the number of cells expressing TSPO based on immunofluorescence do not determine if a change in the number of TSPO binding sites per cell is present. Finally, although evidence is scarce, a direct implication of TSPO on the pathophysiology of the various neuropsychiatric conditions may not be ruled out.

## TSPO expression in human CNS in health, aging, and neurological disease

The cell origin of TSPO according to the human pathology is summarized in Table 2.

**Table 2:** TSPO cell origin in human neurological diseases

Human disease		Microglia	Astrocytes	Neurons	Endothelial cells	Vascular smooth muscle cells	References
Neuroinflammation	Multiple sclerosis	x	x				[7, 169]
Neurodegenerative diseases	Alzheimer’s disease	x	x		x	x	[172, 173]
	Dementia with Lewy-bodies	x					[173]
Infections	HIV encephalitis	x	x	x	x		[167]
	Creutzfeldt-Jakob	x					[178]
Neuropsychiatric disorders	Major depression	x					[184–186]
Stroke		x	x				[167]
Epilepsy		x	x	x			[188]

*HIV* Human immunodeficiency virus

### Health and aging

Little is known of the distribution and expression of TSPO during development, healthy aging, and how such expression differs in regions of the CNS in humans. Consideration of these features, as well as the mode of analysis, e.g. PET, autoradiography, quantitative assays, or pathology of postmortem (PM) brain, is of key importance to explain TSPO expression in PET imaging.

Much of what is known about TSPO expression in humans comes from PET imaging where differential expression may be due to the different affinity patterns for TSPO ligands. PET studies report an increased expression of TSPO with aging in healthy subjects in several cortical and subcortical areas [164–166]. However, little is known about the levels and cellular expression of TSPO during (early) development or in healthy elderly subjects as determined in postmortem control tissues. Quantitative immunoblotting approaches reveal that TSPO protein levels are 2- to 70-fold higher than those reported by in vitro binding assays and expression is widely distributed in the CNS in gray and white matter at all ages [164]. At birth TSPO protein levels are highest in the frontal cortex possibly reflecting expression in neuronal precursor cells although pathology studies have not yet supported this hypothesis. Levels of TSPO decline in the first 3 months after birth and subsequently increase modestly during adulthood/senescence [164]. The relatively high binding and protein expression reported in aging may reflect subtle changes due to senescence or, alternatively, due to changes in the morphology or phenotype of aging cells in the CNS parenchyma. Pathology studies on PM tissues of normal human brains reported that a variety of cell types express TSPO, the levels and extent of expression depending on the TSPO antibody used [167]. Endothelial cells, arachnoid cells, cells within the choroid plexus as well as astrocytes, microglia, and to a lesser degree oligodendrocytes and immune cells within blood vessels revealed a punctate expression typical for mitochondrial expression markers [167]. However, these studies were limited to tissues from aged donors and it is difficult to conclude, based on the available pathology studies, that the cellular expression of TSPO in the normal brain is due to normal aging.

### Neuroinflammation

TSPO PET imaging is widely used to monitor inflammation in MS, a chronic inflammatory demyelinating and neurodegenerative disease with onset in young adults [168]. The PET signal in MS is frequently assumed to represent pathogenic microglia yet pathology studies have detailed a more widespread cellular expression. Compared to normal-appearing white matter in MS tissues where TSPO is expressed in scattered HLA+ cells throughout the CNS, the expression is approximately 20-fold higher in active MS lesions and the rim of chronic active lesions [7]. In addition to microglia, this study revealed that expression is also observed in astrocytes, predominantly in chronic active and inactive lesions, and that the astrocyte signal contributes significantly to the active lesions and rim of chronic active lesions. In addition, this study highlights that TSPO is expressed in some but not all M1 (pathogenic) and M2 (immune-regulatory) phenotypes as well as intermediate microglia/macrophages [169, 170]. Furthermore, a percentage of both TMEM119^+^ and P2RY12^+^ cells, markers that represent homeostatic microglia, expresses TSPO in MS lesions indicating that TSPO PET is not merely a reflection of pathogenic microglia, although the trigger of TSPO upregulation in MS CNS is still unclear. In addition, in MS, TSPO is also expressed by T and B cells in the CNS and thus such cellular expression during disease must be considered in TSPO PET imaging.

### Neurodegenerative diseases

An association between microglia activation, astrogliosis, and neuronal damage has been reported for several neurodegenerative diseases, e.g., AD, Parkinson’s disease, and amyotrophic lateral sclerosis (ALS; motor neuron disease) [171]. As with MS, TSPO PET imaging is widely considered to reflect the pathogenic microglia in neurodegenerative diseases in vivo. Using postmortem brain tissues, several studies have used autoradiography to determine TSPO density in human brain tissues but few have examined the cellular distribution in detail (reviewed by [2]). Recently, a study using immunohistochemistry on AD human brain tissues revealed TSPO expression by microglia, astrocytes, endothelial cells, and vascular smooth muscle cells [172]. TSPO expression was not quantified by cell counts but rather by the amount of TSPO immunoreactivity. Although the authors reported a slight increase of TSPO immunoreactivity in the gray matter compared to healthy subjects, such expression was not associated with Braak stage, Aβ plaques or neurofibrillary tangles or cortical thickness. While these authors showed TSPO expression by CD68 and IBA1^+^ microglia/macrophages, they did not examine expression in specific microglia phenotypes. Receptor density was not found to be increased in postmortem AD brain or in dementia with Lewy bodies (DLB) as investigated with quantitative autoradiography [173]. Interestingly, a significant decrease in receptor density or receptor binding was found in the *substantia nigra* of AD and DLB. No studies have been conducted to investigate the TSPO expression at the cellular level in other neurodegenerative diseases such as ALS, Huntington’s disease, or spinocerebellar atrophy.

### Infections

TSPO expression in the CNS of a few cases with HIV was reported to be similar to healthy human brain [167]. TSPO is reported in metabolic glia, a form of reactive astrocyte, and microglial cells. Tissues from HIV encephalitis (HIVE) cases revealed an increased expression of TSPO in lesioned areas. In cases where the origin of the infection was more unclear, there was a general increase of TSPO expression in activated microglia. HIVE brains showed perivascular TSPO^+^ infiltrates as well as TSPO^+^ microglial nodules and multinucleated giant cells. Studies to determine TSPO expression in infections of the central nervous systems have utilized PET imaging as reported for ZIKA [174] and herpes encephalitis animal models [175–177], as well as Creutzfeldt-Jakob disease patients [178], but have not yet investigated expression in postmortem tissues from humans.

### Neuropsychiatric disorders

Several PET studies of TSPO as a marker of inflammation in psychiatric disorders have been performed but with differing outcomes. For example, PET studies in schizophrenia show different outcomes, either an increase, decrease, or no change compared to controls [179]. A recent review combining several meta-analyses [180–182] showed that overall patients with schizophrenia have lowered TSPO concentrations compared to healthy individuals [183]. On the other hand, in depression, TSPO seems to be upregulated mostly in the anterior cingulate and prefrontal cortex [184–186]. TSPO was overall lower in depression patients receiving SSRI medication compared to unmedicated patients [183]. For bipolar disorder, an increase of TSPO mRNA and protein together with inflammasome activation was found in peripheral blood monocytes [187]. However, while an increasing number of studies show TSPO changes in neuropsychiatric disorders with TSPO PET, there is a paucity of data using human CNS tissues to determine the cellular expression of TSPO in neuropsychiatric disorders to substantiate findings of TSPO PET.

### Stroke

TSPO PET in brain trauma could aid in monitoring regenerative processes after stroke. Depending on the region and severity of the infarct, TSPO is expressed to differing degrees by surrounding microglia and hypertrophic astrocytes. In a subacute infarct in the cerebellar cortex, TSPO^+^ microglia were found to be surrounding/encapsulating Purkinje cells [167].

### Epilepsy

PET studies show increased binding of TSPO ligand in both ipsilateral and contralateral regions in temporal lobe epilepsy (TLE) suggesting inflammation distant to the seizure foci. Examination of brain tissue surgically resected revealed high TSPO expression in microglia and neurons and low expression in astrocytes [188].

## FACS-RTT: a new technique to access to the cellular origin of TSPO

To measure TSPO overexpression, some studies have used histological staining [136, 159]. However, even if this technique presents the advantage of an intact cellular architecture of the tissue, there is not enough quantitative precision to determine the contribution of each cell population of the brain in TSPO signal. Similarly, histological approaches do not allow to assess an important parameter: does an alteration of TSPO in the tissue result from the modulation of the number of cells expressing TSPO? Or does each cell produce more TSPO?

To assess if the overexpression of TSPO is due to a cellular proliferation or an increased expression of TSPO in the cell, an innovative approach was recently developed [189]. This methodology combined the fluorescence-activated cell sorting (FACS) to isolate astrocytes, microglia, neurons, and endothelial cells and the radioligand-mediated labeling of TSPO (RTT, radioligand-treated tissues).

TSPO overexpression was studied in response to acute unilateral injection of lipopolysaccharide (LPS) or of ciliary neurotrophic factor (CNTF), in a rat model of AD and in the AD brain [189, 190]. In all these pathological contexts, TSPO is overexpressed. However, the cellular origin of TSPO overexpression is context-dependent and cellular mechanisms leading to this increase are heterogenous (proliferation of the cell population and/or changes in TSPO expression by each single cell).

Furthermore, the involvement of endothelial TSPO binding in the overall TSPO signal is at the center of in vivo imaging interrogations. In the case of these models of inflammation, endothelial cells contributed to basal TSPO signal but not to its increase [189, 190]. These results potentially answer a fundamental question in the domain of in vivo imaging of neuroinflammation, i.e., regarding the necessity to take the endothelial TSPO signal into account when quantifying TSPO in vivo using PET. Still, it is important to keep in mind that endothelial cells may contribute to TSPO signal in other conditions. Overall, these results confirm the complexity of TSPO and show the necessity to validate their cellular origin in each pathological context before quantifying and interpreting the in vivo imaging signal.

## Conclusion

**Fig. 1:**
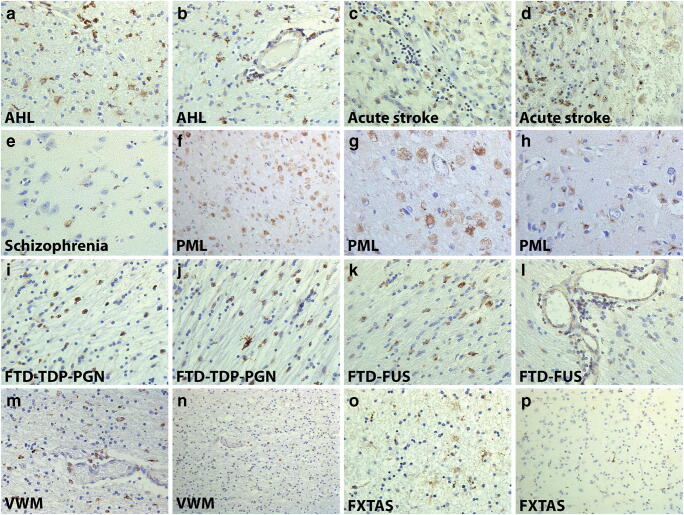
Multiple cell origin of TSPO in the pathological human brain. Astrocytes and perivascular macrophages are positive for TSPO in acute hemorrhagic leukoencephalopathy (A, B). At the site of injury, acute stroke cells express TSPO in two separate cases (C, D). A schizophrenia patient with TSPO+ microglia and endothelial cells in the anterior cingulate cortex (E). Lesions in progressive multifocal leukoencephalopathy are abundant with TSPO in microglia, macrophages, and astrocytes in the white and gray matter (F, G, H). Patients with frontotemporal dementia with mutations for TDP, proganulin, and FUS have a macrophage-like cells expressing TSPO in the white matter and in perivascular spaces (I, J, K, L). Macrophages in vanishing white matter express TSPO throughout the white matter areas in the brain parenchyma (M, N). Fragile X-associated tremor/ataxia syndrome has TSPO+ astrocytes in the white matter and microglia in both white and gray matter (O, P)

Since its identification, TSPO has been widely studied for its different roles in the periphery and more recently within the CNS itself. Its cellular origin demonstrated in the brain in microglia, although it is clear that other cell types also express TSPO and its presence in astrocytes and endothelial cells is now well accepted (Fig. 1). The cellular origin of TSPO alterations likely not only depends on the pathology but also on the developmental stage and mode of cell activation. Monitoring of TSPO levels is now widely used as a marker of inflammation, but research still needs to better characterize the means and cells involved. In this sense, it has recently been reported that the expression of TSPO concerns not only the pro-inflammatory types of microglia but also the anti-inflammatory subtypes. Future studies should also reveal the therapeutic potential of a change in its levels.

## Data Availability

Not applicable.
